# Assessment of Postdonation Outcomes in US Living Kidney Donors Using Publicly Available Data Sets

**DOI:** 10.1001/jamanetworkopen.2019.1851

**Published:** 2019-04-12

**Authors:** Jieming Chen, Sanchita Bhattacharya, Marina Sirota, Sunisa Laiudompitak, Henry Schaefer, Elizabeth Thomson, Jeff Wiser, Minnie M. Sarwal, Atul J. Butte

**Affiliations:** 1 Bakar Computational Health Sciences Institute, University of California, San Francisco; 2 Department of Pediatrics, University of California, San Francisco; 3 Now with the Department of Bioinformatics and Computational Biology, Genentech, Inc, South San Francisco, California; 4 ESAC Inc, Rockville, Maryland; 5 Northrop Grumman Information Systems Health IT, Rockville, Maryland; 6 Division of MultiOrgan Transplant, Department of Surgery and Medicine, University of California, San Francisco

## Abstract

**Importance:**

There are limited resources providing postdonation conditions that can occur in living donors (LDs) of solid-organ transplant. Consequently, it is difficult to visualize and understand possible postdonation outcomes in LDs.

**Objective:**

To assemble an open access resource that is representative of the demographic characteristics in the US national registry, maintained by the Organ Procurement and Transplantation Network and administered by the United Network for Organ Sharing, but contains more follow-up information to help to examine postdonation outcomes in LDs.

**Design, Setting, and Participants:**

Cohort study in which the data for the resource and analyses stemmed from the transplant data set derived from 27 clinical studies from the ImmPort database, which is an open access repository for clinical studies. The studies included data collected from 1963 to 2016. Data from the United Network for Organ Sharing Organ Procurement and Transplantation Network national registry collected from October 1987 to March 2016 were used to determine representativeness. Data analysis took place from June 2016 to May 2018. Data from 20 ImmPort clinical studies (including clinical trials and observational studies) were curated, and a cohort of 11 263 LDs was studied, excluding deceased donors, LDs with 95% or more missing data, and studies without a complete data dictionary. The harmonization process involved the extraction of common features from each clinical study based on categories that included demographic characteristics as well as predonation and postdonation data.

**Main Outcomes and Measures:**

Thirty-six postdonation events were identified, represented, and analyzed via a trajectory network analysis.

**Results:**

The curated data contained 10 869 living kidney donors (median [interquartile range] age, 39 [31-48] years; 6175 [56.8%] women; and 9133 [86.6%] of European descent). A total of 9558 living kidney donors with postdonation data were analyzed. Overall, 1406 LDs (14.7%) had postdonation events. The 4 most common events were hypertension (806 [8.4%]), diabetes (190 [2.0%]), proteinuria (171 [1.8%]), and postoperative ileus (147 [1.5%]). Relatively few events (n = 269) occurred before the 2-year postdonation mark. Of the 1746 events that took place 2 years or more after donation, 1575 (90.2%) were nonsurgical; nonsurgical conditions tended to occur in the wide range of 2 to 40 years after donation (odds ratio, 38.3; 95% CI, 4.12-1956.9).

**Conclusions and Relevance:**

Most events that occurred more than 2 years after donation were nonsurgical and could occur up to 40 years after donation. Findings support the construction of a national registry for long-term monitoring of LDs and confirm the value of secondary reanalysis of clinical studies.

## Key Points

**Question:**

What are the postdonation outcomes of living kidney donors?

**Findings:**

In this cohort study of 10 869 living kidney donors from the ImmPort open access data repository, 9558 individuals’ postdonation data were analyzed. Overall, 1406 living donors (14.7%) had postdonation events; the 4 most common events were hypertension, diabetes, proteinuria, and postoperative ileus, and most events that occurred more than 2 years after transplant were unrelated to surgical complications, occurring up to 40 years later.

**Meaning:**

Aggregated data from publicly available clinical studies can provide insights into short-term and long-term complications affecting living donors.

## Teaser

This cohort study assembles an open access resource that contains more detailed follow-up information to examine postdonation health outcomes for living kidney donors.

## Introduction

Today, solid-organ transplant is the preferred form of treatment for most end-stage organ diseases. While organ donation will benefit the recipients, there are risks and lifelong implications for the living donors (LDs). Annually, approximately 6000 healthy adults in the United States^1^ and 30 000 worldwide^2^ accept the risks of living donation to help family, friends, and strangers. For example, annually between 2007 and 2015, approximately 30 (0.5%) to 50 (0.8%) living kidney donors (LKDs) in the United States developed postoperative kidney failure and entered the organ donation system as potential recipients on the transplant waiting list.^3^ While this rate might not be higher than the general incidence rate for needing a kidney transplant, it is still ideal to be able to manage, minimize, or prevent such morbidities for LDs. In addition, LKDs have been observed to be at increased long-term risk for cardiovascular and end-stage renal disease as well as all-cause mortality compared with matched nondonors who would have been eligible for donation.^4^ Hence, it is necessary to continually improve our understanding of the risks of living donation, including the occurrence of all possible adverse postdonation outcomes.

One of the more prominent gaps in our knowledge of living donation is our ability to visualize and understand the temporal trajectory of long-term postdonation outcomes for LDs, including both end-stage and non–end-stage organ diseases and conditions.^5^ Currently, a database of LDs is maintained by the Organ Procurement and Transplantation Network (OPTN), which is administered by the United Network for Organ Sharing (UNOS).^1^ While these data are quite comprehensive in capturing the breadth of donors, the data collected are largely limited to essential perioperative information for the transplant. Moreover, transplant programs in the United States are required to follow up with the donors for only up to 2 years after transplant.^6^ Even then, there are many barriers to postdonation follow-up.^7^ There has been strong evidence to indicate that ailments can occur many years after donation,^4,8,9^ highlighting the need for more extensive and longer-term data.

ImmPort is an archival repository of clinical research studies funded by the National Institute of Allergy and Infectious Diseases.^10^ As of September 2016, ImmPort contained data from 200 human clinical studies (including 59 clinical trials) in several research foci relating to immunology, including autoimmunity, transplant, and vaccination.^11^ Raw data from clinical studies, especially clinical trials, are typically not easily accessible.^12^ The availability of such data is an invaluable source of LD information, which can be used to visualize clinical study data in organ transplant. ImmPort is also open access and will be updated over time as more clinical studies are submitted by researchers.^10^ We have curated the transplant data sets in ImmPort as an initial proof of concept of its utility and applications, so that the collection, curation, and secondary analyses of other publicly available transplant data can be built on.^13^

## Methods

Deidentified preexisting research data were obtained from the open access repository ImmPort. Since the data made available from ImmPort are only provided as deidentified data, our work did not require the approval of an institutional review board per organizational policy. The UCSF Committee on Human Research issued a research exemption for the deidentified preexisting clinical data used in this study. This study followed the Strengthening the Reporting of Observational Studies in Epidemiology (STROBE) reporting guideline for cohort studies. Methodological details are further explained in the eMethods in the Supplement, and the number of LKDs used in various analyses appears in eTable 1 in the Supplement.

### Study Design

This is a retrospective cohort study of LDs aggregated from the clinical studies found in the ImmPort database. The 27 studies from ImmPort were downloaded September 16, 2016, and included data collected from 1963 to 2016. Our study focused on the most common organ donors, LKDs. Data analysis took place from June 2016 to May 2018.

### Data Sources

#### ImmPort and immTransplant

We used the data release 19 version of the National Institute of Allergy and Infectious Diseases open access data repository ImmPort to identify 27 clinical studies related to solid-organ transplant (study accessions and titles listed in eTable 2 in the Supplement) with a total of 11 263 LDs (eTable 3 in the Supplement). These data contain demographic characteristics as well as perioperative, short-term, and long-term information for LDs across multiple types of transplanted organs (eTable 4 in the Supplement). Not all organs can be transplanted from LDs; typically, kidney, liver, and lung are transplanted from LDs. Because each of these clinical studies has a different study design, set of objectives, and clinical features, we manually curated these studies in a uniform, systematic manner into a standardized data framework that facilitated the extraction of relevant data for visualization and analyses. We curated the data sequentially by study, individual, and record (eFigure 1 in the Supplement). The resultant 20 studies included the Renal and Lung Living Donor Evaluation Study (RELIVE),^8,14,15,16,17,18,19,20^ the Clinical Trial in Organ Transplantation,^21,22,23,24,25,26,27,28,29,30,31^ Coordinated Clinical Trials in Pediatric Transplantation,^32,33,34,35,36,37,38,39,40,41,42^ and other smaller clinical trials.^43,44,45,46,47,48,49^ We referred to our final curated data set as *immTransplant*.^13^ We extracted the LKDs’ race and ethnicity, which are typically self-reported, to assess how representative immTransplant is compared with the national demographic characteristics of LKDs. The racial group definitions from the US Census are usually implemented in clinical studies and, hence, ImmPort studies.

#### UNOS/OPTN National Registry

We obtained data from the UNOS/OPTN national registry. The data files were created in June 2016 and contained information about transplants that occurred in the United States between October 1987 and March 2016. We extracted demographic characteristics and donor-recipient relationship information for LKDs whose transplants took place after October 25, 1999, because records prior to that date have limited medical and demographic fields available. The UNOS/OPTN racial group definitions largely followed the US census, except for the Hispanic/Latino category, which is defined as a racial group by UNOS/OPTN but as an ethnicity in the US Census.^42^ We retained the Hispanic/Latino category as a racial group category in the UNOS/OPTN data set for completeness.

#### Network Construction and Analyses

We obtained 1401 LKD records with 36 documented and dated adverse outcomes from the RELIVE study in immTransplant (eTable 5 in the Supplement). The individuals are LKDs with at least 1 postdonation adverse outcome (LKDOs). For trajectory network construction, each node is an event or condition. The size of the node represents the proportion of LKDOs having that event. We connected 2 events with an edge when both postdonation events occurred for at least 1 LKDO. The network was drawn using Cytoscape (Cytoscape Consortium).^50^

#### Glomerular Filtration Rate Trends in LKDs

We extracted all available predonation and postdonation glomerular filtration rate (GFR) values in immTransplant. Ultimately, we were only able to obtain sufficient numbers for 32 LKDOs with postdonation hypertension. Because we were interested in the overall trend, we categorized an individual as having a GFR decrease if his or her pretransplant GFR measurement was greater than his or her last postdonation GFR measurement, and we categorized an individual as having a GFR increase if his or her pretransplant GFR measurement was lower than his or her last postdonation GFR measurement.

### Statistical Analysis

All Kaplan-Meier analyses were performed and Kaplan-Meier curves plotted using the R version 3.4.4 package survminer (R Project for Statistical Analysis). We obtained right-censored postdonation data from the RELIVE data in immTransplant, which were extracted from the National Death Index. The renal failure end point was defined by any of the following 5 events (whichever comes first for LKDOs with multiple end points): (1) postoperative renal failure, (2) postoperative dialysis, (3) kidney transplant, (4) kidney transplant waiting list, and (5) long-term or maintenance dialysis.

All statistical analyses were performed using statistical software R version 3.4.4 and RStudio (R Foundation) as the integrated development environment. Two-sided Kolmogorov-Smirnov test of heterogeneity, multinomial goodness of fit χ^2^ tests, and Fisher exact tests were implemented using the ks.test, chi.sq, and fisher.test functions, respectively, from the R stats package. *P* ≤ .05 was considered statistically significant in all statistical tests.

## Results

### Demographic Characteristics of immTransplant

There were 10 869 LKDs in immTransplant after curation (Table). These participants had a median (interquartile range) age of 39 (31-48) years, and 6175 (56.8%) were women. The 2 largest racial groups were LKDs of European ancestry (9133 [86.6%]) and black LKDs (1044 [9.9%]). Overall, 3707 LKDs (37.3%) donated their kidneys to their siblings.

**Table.** Living Kidney Donor Characteristics in the ImmPort Database

Living Kidney Donor Characteristic	No. (%)^a^
Data source, No.	10 869
RELIVE	9558 (87.9)
CTOT	863 (7.9)
CCTPT	396 (3.6)
Other	52 (0.5)
Women	6175 (56.8)
Race, No.	10 508
American Indian/Alaskan Native	94 (0.9)
Asian	108 (1.0)
Black	1044 (9.9)
Multiracial	39 (0.4)
Native Hawaiian/Pacific Islander	7 (0.07)
White/European	9133 (86.6)
Other	83 (0.8)
Relationship with recipient, No.	9951
Biologically related	7831 (78.7)
Child	1295 (13.0)
Parent	1925 (19.3)
Sibling	3707 (37.3)
Other	904 (9.1)
Unrelated	2120 (21.3)
Friend	707 (7.1)
Spouse	755 (7.6)
Other	658 (6.6)
Age, median (range) [IQR], y	39 (14-77) [31-48]

Abbreviations: CCTPT, Coordinated Clinical Trials in Pediatric Transplantation; CTOT, Clinical Trial in Organ Transplantation; IQR, interquartile range; RELIVE, Renal and Lung Living Donor Evaluation Study.

^a^ Total number of living donors for each category can differ owing to missing data.

**Figure 1.**
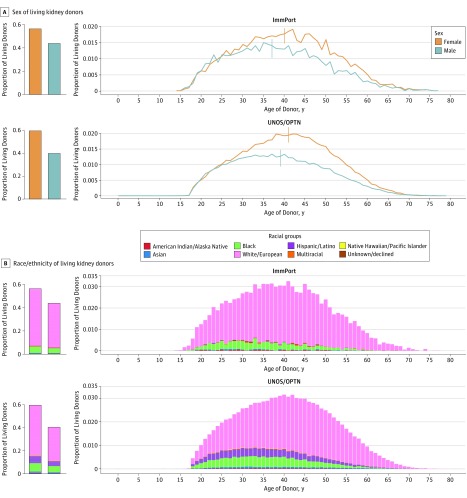
Representativeness of ImmPort Transplant Data With United Network for Organ Sharing/Organ Procurement and Transplantation Network (UNOS/OPTN) Data A, The vertical line indicates the mean age.

To assess the representativeness of our LKD cohort, we compared its sex and race/ethnicity trends with national LKD data from the UNOS/OPTN registry. For this purpose, we extracted the sex and race/ethnicity information from 10 869 LKDs from immTransplant, removing records with missing information (Table). There were 128 407 LKDs in the UNOS/OPTN data set. The sex trends of LKDs in both data sets were very similar, even after stratifying by age of donation (Kolmogorov-Smirnov test of heterogeneity for female donors: D = 0.15; *P* = .36; male donors: D = 0.15; *P* = .45). Overall, there were more female than male LKDs in both data sets, as described previously^51,52,53^ (Figure 1A). However, within each data set, such sex disparity was not uniformly observed across all ages of LKDs. In both data sets, we noticed higher proportions of female LKDs 25 years and older donating to spouses or children (eFigure 2 in the Supplement) compared with male LKDs 25 years and older, whereas there were comparable proportions of female and male LKDs younger than 25 years (Figure 1A; eFigure 3 in the Supplement).

Figure 1B shows that for both immTransplant and UNOS/OPTN data, most LKDs were white followed by black donors (multinomial goodness-of-fit χ^2^ test: χ^2^_36_ = 42; *P* = .23). Also, in both data sets, we observed biases for female donors and age groups 26 to 35 years and 36 to 45 years across racial groups with 100 or more LKDs (eFigure 4 in the Supplement). Owing to differences in the definitions of the various categories of race and ethnicity in the 2 data sets, the ImmPort data set does not contain Hispanic/Latino—the third largest racial category in the UNOS/OPTN data set—as a racial category. Otherwise, the ethnic distributions in the 2 data sets were very similar. Taken together, these analyses indicate that curated LD data in ImmPort is a good representation of the national transplant data in terms of sex, race/ethnicity, and age.

### Postdonation Outcome Trajectory Network for LKDs

Next, we examined the data from postdonation conditions in LKDs, which originate from the RELIVE data set and contain 9558 LKDs. Most (8152 of 9558 [85.3%]) LKDs did not have recorded outcomes (eTable 1 in the Supplement), which could be owing to an inability to follow up or to a lack of adverse outcomes. Overall, 1406 LKDs (14.7%) had postdonation events. We focused on the 1401 LKDs with at least 1 of 36 postdonation outcomes (eTable 5 in the Supplement). Some of the postdonation outcome events that affected the highest number of LKDOs were cardiovascular or kidney-related conditions (eTable 5 in the Supplement). Hypertension affected the most LKDOs (806 [8.4%]), followed by diabetes (190 [2.0%]), proteinuria (171 [1.8%]), and postoperative ileus (147 [1.5%]) (eTable 5 in the Supplement). Although LKDs with postdonation hypertension constituted 806 of 1401 LKDOs (57.4%) in our data set, hypertension only corresponds to an overall occurrence rate of 8.4% in the entire LKD data set, which includes those with no postdonation conditions (eTable 5 in the Supplement).

**Figure 2.**
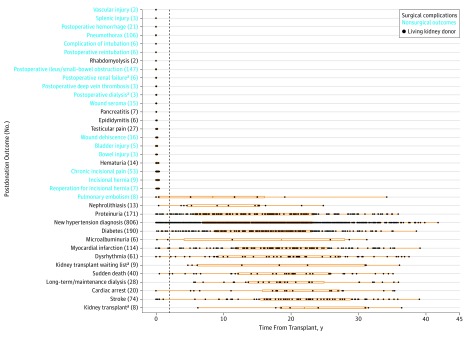
Time of Diagnoses of Postdonation Outcomes of Living Donors in the Renal and Lung Living Donor Evaluation Study Ends of orange box plots represent the first (leftmost end of box) and third (rightmost end of box) quartiles. Conditions on the y-axis are arranged in increasing order of median number of years of diagnosis, represented by the middle of the box plot (50th percentile). The vertical dotted line indicates the 2-year postdonation mark. ^a^Conditions associated with renal failure.

Overall, 269 and 1746 events occurred before and after the 2-year postdonation mark, respectively. We further classified the outcomes into postoperation surgical complications and nonsurgical conditions (eTable 5 in the Supplement). Most conditions (248 of 269 [92.2%]) that occurred 2 years or earlier after transplant were surgical complications, whereas 1575 of 1746 conditions (90.2%) that occurred 2 years or more after transplant were nonsurgical. Nonsurgical conditions tended to occur in the wide range of 2 to 40 years after donation (odds ratio, 38.3; 95% CI, 4.12-1956.9; Fisher exact test of *P* < .001) and could take a median of more than 5 years to surface (Figure 2).

**Figure 3.**
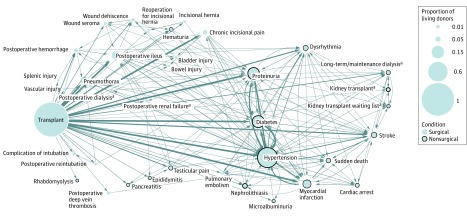
Trajectory Network of Postdonation Outcomes of Living Donors in Renal and Lung Living Donor Evaluation Study Relative lengths of edges not drawn to scale. Nodes ordered horizontally according to the mean time of diagnosis. Edge thickness indicates the number of living kidney donors who have event X followed by event Y. Five conditions are associated with renal failure. ^a^Conditions associated with renal failure.

With temporal information, we were able to construct a trajectory map of postdonation outcomes for visualization (Figure 3). These outcomes included end-stage renal disease and non–end-stage renal disease outcomes. By further defining events for renal failure, we were able to visualize that not all 33 possible outcomes are intermediate events leading up to renal failure. Only some of the largest intermediate nodes are cardiovascular and kidney-related events, which are known to precede renal failure^54,55,56^ (Figure 3; eTable 5 in the Supplement).

In addition to providing a visual representation, the network further enabled us to examine the number and type of postdonation events occurring in only the LKDOs. We observed that 991 of 1401 postdonation conditions (70.7%) occurred singly in LKDOs, with 12 main cardiovascular and kidney-related events making up 689 of 991 of these single events (69.5%) (eFigure 5 in the Supplement). This suggests that LKDs, in general, can experience renal or cardiovascular events that increase their likelihood of renal failure without first experiencing intermediate events. It also implies that the development of earlier postdonation complications or conditions is not necessarily indicative of long-term renal function or vice versa. Taken together, these observations strongly support the need for long-term systematic renal health monitoring and routine regular checkups for LKDs.

### Postdonation GFR Trends in LKDs

We then further examined GFR measurement trends in LKDs before and after donation. Because of insufficient numbers, we were only able to report results for LKDOs on the most common single outcome of the 11 conditions we investigated (postdonation hypertension) (eMethods and eFigure 6 in the Supplement). For most LKDOs with postdonation hypertension (25 of 32 [78%]), GFR decreased from pretransplant healthy levels (eFigure 6 in the Supplement). In fact, GFR levels decreased below 60 mL/min/1.73 m^2^ in 18 LKDOs (56%), which is the threshold that indicates moderate kidney disease as defined in the 2012 Kidney Disease Improving Global Outcomes guidelines.^57^

### Kaplan-Meier Analyses

**Figure 4.**
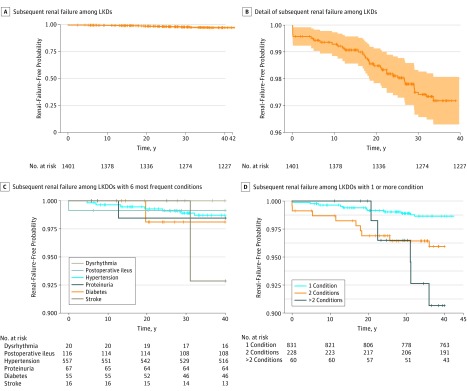
Kaplan-Meier Analyses of Renal-Failure–Free Probability in Living Kidney Donors (LKDs) With 1 or More Adverse Outcomes Each cross on the line represents a right-censored data point. A and B, The area above and below the curve indicate the 95% CIs. B, The panel shows a detail view of the data presented in panel A. C and D, LKDO indicates living kidney donor with 1 or more adverse outcomes.

Finally, we investigated whether the type and number of postdonation conditions gave rise to differences in event-free rates for renal failure (or renal-failure–free probability, with renal failure as defined in the network analysis) by performing Kaplan-Meier analyses on the LKDOs. We first observed that the occurrence of subsequent renal failure among LKDOs was generally low, with an overall event-free rate of 97% when we included LKDs with no recorded outcomes (Figure 4A and Figure 4B). Then, by restricting renal-failure–free analysis to the 6 most frequently occurring single conditions among LKDs (ie, hypertension, proteinuria, diabetes, myocardial infarction, dysrhythmia, and stroke) (eFigure 5 in the Supplement), we found that LKDOs with stroke, which can arise decades after transplant, seemed to be associated with the least favorable outcomes. Not surprisingly, the only 2 nonsurgical complications, dysrhythmia and postoperative ileus, had the most favorable outcomes, as they arose only within the first year of transplant (Figure 4C). These conditions could also occur singly in each LKDO or as one of a number of conditions occurring in a single LKDO. We found that LKDOs with more of these conditions appeared to be associated with less favorable outcomes apropos of having renal failure (Figure 4D).

## Discussion

In June 2016, the White House organized a summit to increase organ donation and decrease the size of the transplant wait list.^58^ One of the primary initiatives included plans to establish a living donor registry to improve recruitment, awareness, education, research, and long-term health management of potential LDs. Apart from the UNOS/OPTN registry database, to our knowledge, there is currently no unified and centralized resource of living donation dedicated and openly available to the transplant research community and the general public. There are a variety of reasons for the difficulty of obtaining such data. For example, many LDs are generally not adherent in following up with appointments, especially when many of them do not develop major complications within the first few years after transplant. While the UNOS/OPTN registry database is a premier data resource, there are challenges in using it. For example, gaining access to the database requires an application process. Additionally, the registry currently places more emphasis on acquiring recipient than LD information. Hence, building a central repository for LDs would require a more systematic approach that has to be established at the national level, where recent foundational efforts are finally underway by the Living Donor Collective.^59^

Our current work serves to supplement existing resources to support ongoing LD research while such a nationwide LD registry is being established. We took a complementary step by carefully curating an online open access resource for LD data.^13^ We created a representative database that is uniformly curated from open access clinical studies from ImmPort. We have shown the utility of the data in a variety of ways. We have visualized donation demographic characteristics in a way that can provide insights into donation patterns and potential strategies to better inform LDs. For example, we found a spike in living kidney donations among women around the range of childbearing years (25 years)^60^ in the United States. Because it has been shown that kidney donation increases the risks of hypertension and preeclampsia in pregnancies,^5,61,62^ LDs in the childbearing age range should be better informed and counseled. Using our data, we were able to visualize the postdonation outcome data by creating a trajectory map, which integrates surgical and nonsurgical postdonation outcome data with temporal information. By further coupling with survival analyses and GFR measurement trend analyses, we can better understand long-term postdonation outcomes and their association with the possibility and risk of organ failure in LDs. Our postdonation outcome analyses also strongly suggest that longer mandatory follow-up periods for LDs will help us understand the long-term effects and sequence of events after transplant. Overall, our work sets a blueprint for collection, analysis, and open-source sharing of donor data and also highlights the importance of long-term follow-up of LDs.

### Limitations

The main limitation of our study is the lack of long-term follow-up information for most patients in the RELIVE cohorts. Only a subset of the surviving RELIVE LKDs were presented with questionnaires for follow-up surveys on their postdonation outcomes. Consequently, we could not determine definitely whether the lack of record was owing to not having a given event or to missing follow-up data; missing data is a common issue in clinical studies. As such, there exists a risk of selection bias, especially in computations that involve LKDs with no recorded outcome, such as the overall rates of occurrence and survival. Caution should be taken when interpreting these results. More follow-up data are needed to improve the interpretation of results.

The current study also bears other limitations. First, the single RELIVE consortium constitutes most of our data. The Renal and Lung Living Donors Evaluation Study is a retrospective cohort study from 3 transplant centers, which collected donor and recipient information from medical records and national databases. Most of the original RELIVE studies focused on several aspects that were not necessarily related to the postdonation outcomes of kidney and lung LDs, such as metabolic and blood pressure profiles,^14^ health-related quality of life,^17^ satisfaction with life,^19^ emotional well-being,^16^ emotional and financial experiences,^15^ and informed consent issues.^20^ Consequently, our analyses are essentially secondary reanalyses of the RELIVE data. Previous secondary reanalyses of open access clinical studies have already been shown to uncover new or reinforce old biological and/or clinical insights and knowledge.^63,64^ While our orthogonal use of the open access clinical studies from the original RELIVE study does show the power of secondary reanalyses, it would definitely be more beneficial to combine a higher diversity of similarly sized clinical studies. As more clinical studies are constantly being added to ImmPort, this issue can potentially be alleviated over time. Second, we did not adjust for potential confounders that exist owing to demographic or clinical characteristics.

## Conclusions

In addition to research, LD resources serve as an educational platform to disseminate knowledge of living donation to the general public. Such dissemination may, in turn, perpetuate a more well-informed discourse for living organ donation between potential LDs, recipients, clinicians, researchers, and the rest of the public. Conceivably, we would also like to raise awareness among recipients, LDs, and health care professionals of the need to consider long-term monitoring for LDs. Current assays in clinical development, such as the Kidney Injury Test (KIT), which can accurately detect kidney injury in the residual donor kidney, may be useful for monitoring and improving overall health outcomes.^65^ Ultimately, we want to encourage and empower potential LDs, not only in seeing the benefits of living donation in saving the lives of their loved ones and strangers but also in better understanding the risks and making more informed choices when deciding to become living donors.

## Notes

Supplement. **eMethods.** Details of Methods section **eTable 1.** LKDs used in various analyses **eTable 2.** 27 clinical studies in ImmPort that are related to transplantation **eTable 3.** Living donor characteristics in the immTransplant curated data set **eTable 4.** Categories of nonclinical and clinical features **eTable 5.** 36 postdonation outcomes found in the LKDs in ImmPort RELIVE **eFigure 1.** ImmPort transplant data curation workflow **eFigure 2.** Increase in female donations at age >25 years mainly due to donations to offspring and spouses **eFigure 3.** Higher proportions of female LKDs at age of donation >25 years **eFigure 4.** Sex and age group bias in immTransplant and UNOS/OPTN data sets **eFigure 5.** Number and type of posttransplant conditions that can occur in each LKD **eFigure 6.** GFR values decrease after donation in living kidney donors
