# Error in Figure 2

**DOI:** 10.1001/jamanetworkopen.2019.8822

**Published:** 2019-07-17

In the Original Investigation titled “Assessment of Postdonation Outcomes in US Living Kidney Donors Using Publicly Available Data Sets,”^1^ published April 12, 2019, there was an error in Figure 2. The key should have listed surgical complications in blue text and nonsurgical complications in black text. This article has been corrected.^1^
