# Supplementary material for: Assessment of Postdonation Outcomes in US Living Kidney Donors Using Publicly Available Data Sets
Source: JAMA Netw Open. 2019 Apr 12;2(4):e191851. doi: 10.1001/jamanetworkopen.2019.1851 (PMC6481454; doi:10.1001/jamanetworkopen.2019.1851)
Supplement: Supplement. — eMethods. Details of Methods section eTable 1. LKDs used in various analyses eTable 2. 27 clinical studies in ImmPort that are related to transplantation eTable 3. Living donor characteristics in the immTransplant curated data set eTable 4. Categories of nonclinical and clinical features eTable 5. 36 postdonation outcomes found in the LKDs in ImmPort RELIVE eFigure 1. ImmPort transplant data curation workflow eFigure 2. Increase in female donations at age >25 years mainly due to donations to offspring and spouses eFigure 3. Higher proportions of female LKDs at age of donation >25 years eFigure 4. Sex and age group bias in immTransplant and UNOS/OPTN data sets eFigure 5. Number and type of posttransplant conditions that can occur in each LKD eFigure 6. GFR values decrease after donation in living kidney donors [file jamanetwopen-2-e191851-s001.pdf]

Supplementary Online Content

Chen J, Bhattacharya S, Sirota M, et al. Assessment of postdonation outcomes in US living kidney donors using publicly available data sets. *JAMA Netw Open*. 2019;2(4):e191851. doi:10.1001/jamanetworkopen.2019.1851

eMethods. Details of Methods section
eTable 1. LKDs used in various analyses
eTable 2. 27 clinical studies in ImmPort that are related to transplantation
eTable 3. Living donor characteristics in the immTransplant curated data set
eTable 4. Categories of nonclinical and clinical features
eTable 5. 36 postdonation outcomes found in the LKDs in ImmPort RELIVE
eFigure 1. ImmPort transplant data curation workflow
eFigure 2. Increase in female donations at age >25 years mainly due to donations to offspring and spouses
eFigure 3. Higher proportions of female LKDs at age of donation >25 years
eFigure 4. Sex and age group bias in immTransplant and UNOS/OPTN data sets
eFigure 5. Number and type of posttransplant conditions that can occur in each LKD
eFigure 6. GFR values decrease after donation in living kidney donors

This supplementary material has been provided by the authors to give readers additional information about their work.

eMethods. Details of Methods section

This section gives in detail the methods used in this work.

Curating the ImmPort transplant data to create immTransplant
We curated the data sequentially by study, subject and at the record level.

(1) At the study level, we first identified 27 ImmPort clinical studies related to solid organ transplantation and obtained their data dictionaries and case report forms (CRFs) (ImmPort version DR19, accessed September 16, 2016), for a total of 11,263 LDs. Then, we removed five studies that explicitly excluded living donation, and one study that did not have a sufficiently complete data dictionary in ImmPort. At the study level, even though there were more recipient-centric studies, 88% of the LDs are contributed by the living-donor-centric RELIVE studies (eFigure 1).

(2) The resultant 20 studies (12,354 subjects) were subsequently curated by subjects. We removed 928 deceased donors, and 162 living donors with > 95% missing data. Because some subjects could be involved in multiple studies, we also merged their redundant records, resulting in 11,263 living donors.

(3) Finally, the data in this cohort was compiled and classified into five main categories, namely (A) demographics, (B) donor relationship to recipient, and (C) pre-transplant, (D) intraoperative, and (E) post-transplant data (eTable 5). We then standardized the data in the fields that were common across studies, in terms of units of measurement and definitions. For example, we converted all units for weight measurements to the SI unit of kilogram and centimeter for height. In terms of definitions in common fields, the same data representation in two clinical studies could have different meanings. For example, in a field that coded for the donor's relationship to the recipient in two studies, a '2' can denote 'parent-child' in one study but 'siblings' in another. Hence, when merging across studies, we converted all numeric entries to English words, as defined in the respective data dictionaries, and standardized synonymous English words. For the fields that were both unique to a study (due to the design of a clinical study), and also fell into one of the five aforementioned categories, we added new columns for these fields and tag the empty records as missing ('NA').

The consolidated data is available at http://www.immport.org/resources/TTN. The data includes all the processed clinical study files that are used in this manuscript from ImmPort for solid transplantation. We also included a Cytoscape file of the trajectory map for user visualization and download.

Demographic and relationship information from immTransplant
We extracted the gender and racial information from 10,869 LKDs and removed records with missing information. For trend comparisons, we used 10,833 LKDs with age at donation and gender, and 10,494 with age and racial information. For the relationship analysis, we used 9,526 LKDs with age at donation and the donor's relationship to the recipient. For racial group comparisons with UNOS/OPTN, we only considered the three racial groups with insufficient individuals for reasonable comparisons (an arbitrarily chosen threshold of 100 individuals), namely Caucasian, African, and Asian American. Note that the proportions in eFigure 4 were calculated based on the total number of LKDs within each racial group

Assessment of representativeness for immTransplant
In order to demonstrate that the entire *immTransplant* data is representative of the national data demographically for downstream analyses, we compared the demographic trends of all the LKDs in *immTransplant* versus the U.S. UNOS/OPTN national registry. There were 128,407 in the UNOS/OPTN dataset. Overall, there are more female than male LKDs in both datasets; such gender disparity in LKDs has been described previously[52–54] (Figure 1A). Even as we further stratified the proportion of male and female LKDs by their ages at kidney donation, we still observed that both the female and male LKD distributions in *immTransplant* and the UNOS/OPTN dataset are very similar (Kolmogorov–Smirnov test of heterogeneity, D = 0.15, non-significant p = 0.36 for female; D = 0.15, p = 0.45 for male).

However, within each dataset, a higher proportion of female LKDs was not uniformly observed across all ages of LKDs. In both *immTransplant* and UNOS/OPTN, we noticed higher proportions of female LKDs relative to male at

© 2019 Chen J et al. *JAMA Network Open.*

ages over 25 years, whereas we observed comparable proportions of female and male LKDs who donated at ages less than 25 years old (Figure 1A and eFigure 3). Because the age of 25 is the mean age of childbearing mothers in the U.S.,[55] we further investigated this gender disproportionality of donors, by examining the donors' relationships to the recipient. Specifically, we explored whether there are any trends between the gender distributions of donors in terms of their relationship to the recipients, who are less or more than 25 years of age (eFigure 2). In both *immTransplant* and UNOS/OPTN data, relatively more women (than men) donate kidneys after age 25 (as compared to LKDs age ≤ 25), preferentially to their spouses or offspring (eFigure 2).

Network construction and analyses

We obtained 1,406 LKDs, documented with at least one post-transplant adverse outcome (LKDOs). We further removed records that had no date associated with the outcome, and those in which the initial diagnosis already occurred before transplant. Finally, we used 1,401 LKDOs with 36 documented outcomes (eTable 4).

For trajectory network construction, each node (circle) is an event, or condition. The size of a node represents the proportion of LKDOs having that event (please refer to eTable 4 for actual numbers). We connect two events with an edge when both post-donation events occur for at least one LKDO. The network is drawn using the software, Cytoscape.[42]

Since the two events can be ordered according to the time it first occurs after transplant, we connect the two events by a directed edge (arrow), such that an event X precedes an event Y in at least one LKDO's post-donation timeline. We started with the time of transplantation as the leftmost black node. The thickness of the edge represents the number of LKDOs having that trajectory from event X to Y. The nodes are ordered on the horizontal axis according to the mean time of initial diagnosis, but the relative positions are not drawn to scale, i.e. the length of the edges are not necessarily scaled.

Glomerular filtration rate trends in LKDs

We imposed the following criteria on the 1,401 LKDOs used in the trajectory map and 8,152 LKDs with no conditions in RELIVE: (1) we retained an individual, if and only if, we have one pre-transplant GFR value as well as at least one post-transplant value with non-missing dates; (2) we assumed all missing dates for pre-transplant GFR measurement (those marked as 'NA') were performed on the day of transplant, hence these were set to 0; (3) we removed an individual if the pre-transplant GFR value was obtained on a date (number of days) after transplant (as denoted by a positive value in the date that the GFR was measured); (4) we removed an individual if all the post-transplant GFR values were recorded before the transplantation (denoted by a negative value in the date for GFR measurement). These filters resulted in 32 LKDOs with post-transplant hypertension and 75 LKDs with no conditions; the rest of the conditions have less than 10 individuals.

Kaplan-Meier analyses

All Kaplan-Meier analyses were performed, and Kaplan-Meier curves plotted using the R package *survminer*. We obtained right-censored data from the RELIVE data in *immTransplant* that were extracted from the National Death Index. The 'renal failure' endpoint is defined by either of the five events, whichever comes first in LKDOs with multiple endpoints: "post-operative renal failure", "post-operative dialysis", "kidney transplant", "kidney transplant waiting list" and "chronic/maintenance dialysis". For dates, a positive value indicates number of days post-transplant, while a negative value indicates number of days pre-transplant, and the day of transplant is '0'; 'days' are then converted to 'years'. 1,401 LKDOs are used to analyze overall event-free 'survival', or renal-failure-free probability, among LKDOs. For analyses in Figure 4, we stratify the *immTransplant* data to visualize the renal-failure-free survival of LKDOs for three scenarios: (1) the entire LKDOs sample, (2) among the LKDOs stratified by the five most frequently occurring conditions in our dataset (i.e. the five largest nodes in the trajectory network), and (3) among the LKDOs in (2) but stratified by the number of conditions they have. We also assess the survival of 831 LKDOs with only a single condition from the six most frequently occurring single conditions in the dataset (diabetes, dysrhythmia, hypertension, post-operative ileus, proteinuria, stroke); or 1,120 if we consider additionally LKDOs with at least one of the six conditions. We marked right-censored data points as individuals who died without renal failure before 40 years post-transplant.

Statistical Analyses

© 2019 Chen J et al. *JAMA Network Open.*

All statistical analyses were performed using statistical software, R, and RStudio as the integrated development environment. The Kolmogorov-Smirnov (KS) tests (two-sided) were implemented using the ‘ks.test’ function in the ‘stats’ R package. The KS test is a non-parametric test to compare the cumulative distribution functions between a sample and a reference distribution. We included the D statistic from the KS test as it measures the absolute maximum distances between the two cumulative distributions. The multinomial goodness of fit chi-squared test was implemented using the ‘chisq.test’ function in the ‘stats’ R package. We have also included the chi-square statistic ($\chi 2$) and the degree of freedom (df). The Fisher’s exact test was implemented using the ‘fisher.test’ function in the ‘stats’ R package. We included the odds ratio as an estimate for the effect size. A p value cutoff of 0.05 (and below) is considered statistically significant in all statistical tests.

© 2019 Chen J et al. *JAMA Network Open.*

eTable 1. LKDs used in various analyses

Description	Number of LKDs	% LKDs
**Representativeness analysis**		
Total number of LKDs in *immTransplant* (including all the clinical studies)	10,869	100%
Number of LKDs in *immTransplant* with racial information	10,508	96.7%
Number of LKDs in *immTransplant* with donor-recipient relationship information	9,951	91.6%
**Trajectory network analysis**		
Total number of LKDs in RELIVE	9,558	100%
Number of LKDs with no recorded outcomes in RELIVE	8,152	85.29%
Number of LKDs with at least one recorded outcome in RELIVE but has no time of diagnosis associated with the outcomes	5	0.05%
Number of donors with at least one recorded outcome in RELIVE and contains a time of diagnosis associated with at least one outcome (LKDOs)	1,401	14.66%
**GFR trends**		
**LKDOs**		
Total number of LKDOs in RELIVE	1,401	100%
Number of LKDOs in RELIVE with pre- and post-transplant GFR values, and with post-transplant hypertension	32	2.3%
**LKDs with no outcomes**		
Total number of LKDs with no recorded outcomes in RELIVE	8,152	100%
Number of LKDs with no recorded outcomes in RELIVE, and with pre- and post-transplant GFR values	75	0.9%
**Kaplan-Meier analyses**		
Total number of LKDOs in RELIVE	1,401	100%
Number of LKDOs in RELIVE with only a single of the following condition: hypertension, proteinuria,	831	59.3%

© 2019 Chen J et al. *JAMA Network Open.*

diabetes, myocardial infarction, dysrhythmia, and stroke		
Number of LKDOs in RELIVE with at least 1 of the following conditions: hypertension, proteinuria, diabetes, myocardial infarction, dysrhythmia, and stroke	1,120	79.9%

This table shows the breakdown of LKDs used in the various analyses, based on the entire *immTransplant* curated dataset or the subset of just the RELIVE studies, as described in the first column. The last column shows the percentage of the LKDs in each respective analysis. GFR, glomerular filtration rate; LKD, living kidney donor; LKDO, living kidney donor with outcome (at least one)

© 2019 Chen J et al. *JAMA Network Open.*

eTable 2. 27 clinical studies in ImmPort that are related to transplantation

#	ImmPort Study ID	ClinicalTrials.gov ID	Number of living donors with data	Organ	PubMed IDs	Cohort	ImmTx (Y/N)
1	SDY131	NCT00023231	33	kidney	16382014, 16687625, 15049798, 21538345	CCTPT	Y
2	SDY132	NCT00141037	52	kidney	23009139, 22694755, 22694733	CCTPT	Y
3	SDY133	NCT00023244	163	kidney	19663893, 18416737, 19958331	CCTPT	Y
4	SDY289	NCT00608283	8,922	kidney	23137211, 26463883, 25293374, 24011252, 21564530, 25136843	RELIVE	Y
5	SDY290	NCT00951977	98	kidney	24011252, 21564530, 25136843, 24011252, 21564530, 25136843	RELIVE	Y
6	SDY291	NCT01158742	413	kidney	24011252, 21564530, 25136843, 24011252, 21564530, 25136843	RELIVE	Y
7	SDY292	NCT01742234	636	kidney	21158924	RELIVE	Y
8	SDY293	NCT00553397	369	lung	25039865	RELIVE	Y
9	SDY294	NCT01524835	162	lung	25039865	RELIVE	Y
10	SDY352	NCT00047983	54	kidney	NA	CCTPT	Y
11	SDY356	NCT00090194	18	kidney	NA	CCTPT	Y
12	SDY358	NCT00005113	76	kidney	NA	CCTPT	Y
13	SDY479	NCT00337220	273	kidney	23822777, 26047788	CTOT	Y
14	SDY546	NCT01338779	29	kidney	22151236, 20501946, 26461968	--	Y
15	SDY557	NCT00308802	192	kidney	23763485, 26226830, 23802725, 23968332, 24568099, 23710568	CTOT	Y
16	SDY662	NCT00320606	25	liver	22253395	--	Y
17	SDY668	NCT00183248	9	kidney	NA	--	Y
18	SDY671	NCT00078559	9	kidney	19344431, 26461968	--	Y
19	SDY674	NCT00346151	5	kidney	16120857	--	Y
20	SDY689	NCT00307125	398	kidney	NA	--	Y
21	SDY134	NCT00240994	34	kidney	22052056	CCTPT	N
22	SDY354	NCT00000936	0	kidney	NA	CCTPT	N
23	SDY355	NCT00000935	0	kidney	NA	CCTPT	N
24	SDY357	NCT00005009	0	kidney	NA	CCTPT	N
25	SDY567	NCT00014911	36	islet	17005949, 15257060	--	N
26	SDY571	NCT00466804	0	heart	23763485, 26260101,30010623	CTOT	N
27	SDY670	NCT00531921	0	kidney	23710539, 20677285, 28117940	CTOT	N

© 2019 Chen J et al. *JAMA Network Open.*

Among them, 20 studies are included in our analyses, while 7 studies are excluded, due to lack of information for living donors (reasons include exclusion of living donations, insufficient data on living donors, or incomplete data dictionary). We also focus only on solid organ transplantation, hence SDY567 is excluded, since it pertains to stem cell transplantation. We obtain the PubMed IDs for those studies that have been published; those clinical studies without publications are marked 'NA'. The studies are mainly from the Renal and Lung Living Donor Evaluation Study (RELIVE), the Clinical Trial in Organ Transplantation (CTOT) and Coordinated Clinical Trials in Pediatric Transplantation (CCTPT).

© 2019 Chen J et al. *JAMA Network Open.*

eTable 3. Living donor characteristics in the immTransplant curated data set

Living donor characteristic	Number of living donors* (%)
Data source	11,263
RELIVE	9,927 (88.1)
CTOT	863 (7.7)
CCTPT	396 (3.5)
Others	77 (0.7)
Organ	11,263
Kidney	10,869 (96.5)
Lung	369 (3.3)
Liver	25 (0.2)
Female	6,329 (56.2)
Race	10,895
African/African American	1,051 (9.6)
American Indian/Alaskan Native	94 (0.9)
Asian	114 (1.0)
Multiracial	40 (0.4)
Native Hawaiian/Pacific Islander	8 (0.07)
White/European	9,505 (87.0)
Others	83 (0.8)
Relationship with recipient	10,313
Biologically related	8,095 (78.5)
Child	1,297 (12.6)
Parent	2,034 (20.0)
Sibling	3,768 (36.5)
Others	996 (9.7)
Unrelated	2,218 (21.5)
Friend	707 (6.9)
Spouse	755 (7.3)
Others	675 (6.5)
Median age	39

*Please note that the total number of living donors for each category can differ due to missing data.

This table summarizes the living donor characteristics in the ImmPort database.

© 2019 Chen J et al. *JAMA Network Open.*

eTable 4. Categories of nonclinical and clinical features

Non-clinical
**Demographics**
Gender<br>Ethnicity<br>Race<br>Education level<br>Marital Status<br>Religion<br>Age at transplant<br>Employment status<br>Disability<br>Insurance coverage
**Recipient information**
Recipient’s relationship to donor
Clinical
**Pre-transplant information**
Physical examination<br>- including weight, height, blood pressure, cholesterol levels, white and red blood cell counts<br>- *For kidney*: glomerular filtration rate (GFR), creatinine clearance, urine protein test<br>- *For lungs*: lungs ventilation and perfusion (VQ) proportion, lung volumes, blood gas evaluation<br>Human Leukocyte Antigen status (HLA)<br>Blood type<br>Serology<br>Medical history
**Intraoperative information**
Type of surgical incision<br>Surgical procedure<br>Length of time stayed in intensive care unit (ICU)<br>Some surgical details:<br>- *For kidney*: left/right kidney was removed, laparoscopic or open surgery, estimated blood loss, other incidental surgical procedure<br>- *For lungs*: which lobe was donated, stump handling bronchoplastic procedure, intubation
**Post-transplant information**
Physical examination<br>- including weight, height, blood pressure, cholesterol levels, white and red blood cell counts, post-operative infections<br>- *For kidney*: GFR, creatinine clearance, fasting glucose levels, urine protein test, iothalamate concentration<br>- *For lungs*: bronchodilator measurements, spirometry measurements<br>Outcomes<br>- Including time from transplant till death, causes of death<br>- *For kidney*: onset of the following events/conditions – epididymitis, reintubation, extubation, cardiac arrest, stroke, embolism, myocardial infarction, bladder injury, bowel injury, splenic injury, vascular injury, wound dehiscence, wound seroma, pneumothorax, hernia, chronic incisional pain, testicular pain, pancreatitis, rhabdomyolysis, post-operative renal failure, post-operative dialysis, post-operative deep vein thrombolism (DVT), post-operative hemorrhage, post-operative ileus, hematuria, microalbuminuria, proteinuria, nephrolithiasis, dysrhythmia, diabetes mellitus, hypertension, chronic dialysis, kidney transplant<br>- *For lung*: onset of the following events/conditions – extubation, pleural effusion, hemothorax, pneumothorax, pneumonia, empyema, wound infection, urinary tract infection, phlebitis,

© 2019 Chen J et al. *JAMA Network Open.*

	clostridium difficile colitis, breast implant rupture, anesthetic complication, thrombosis, air embolism, ileus, wound seroma, wound dehiscence, myocardial infarction, post-operative arrhythmia, pericarditis, paralyzed hemidiaphragm injury, peripheral nerve injury, pulmonary artery/vein thrombosis, DVT, brachial plexus injury, atelectasis, stenosis, reoperation, bronchoscopy, acute respiratory illness, chronic obstructive pulmonary disease (COPD), chronic bronchitis

The demographics and recipient categories contain non-clinical information about the living donors, while the pre-, intra- and post-operative categories contain clinical information.

© 2019 Chen J et al. *JAMA Network Open.*

eTable 5. 36 postdonation outcomes found in the LKDs in ImmPort RELIVE

Rank	Post-donation condition	Number of LKDs	% (out of 1,401 LKDs)	Occurrence Rate (% out of all 9,558 LKDs)	Surgical complication/Non-surgical condition (S/NS)
1	New Hypertension Diagnosis (post-transplant)	806	57.53	8.43	NS
2	Diabetes	190	13.56	1.99	NS
3	Proteinuria	171	12.21	1.79	NS
4	Post-operative Ileus/SBO	147	10.49	1.54	S
5	Myocardial Infarction	114	8.14	1.19	NS
6	Pneumothorax	106	7.57	1.11	S
7	Stroke	74	5.28	0.77	NS
8	Dysrhythmia	61	4.35	0.64	NS
9	Chronic Incisional Pain	53	3.78	0.55	S
10	Sudden Death	40	2.86	0.42	NS
11	Chronic/Maintenance Dialysis	28	2.00	0.29	NS
12	Testicular Pain	27	1.93	0.28	NS
13	Post-operative Hemorrhage	21	1.50	0.22	S
14	Cardiac Arrest	20	1.43	0.21	NS
15	Wound Dehiscence	16	1.14	0.17	S
16	Wound Seroma	15	1.07	0.16	S
17	Hematuria	14	1.00	0.15	NS
18	Nephrolithiasis	13	0.93	0.14	NS
19	Incisional Hernia	9	0.64	0.09	S
20	Kidney transplant waiting list	9	0.64	0.09	NS
21	Kidney transplant	8	0.57	0.08	NS
22	Pulmonary Embolism	8	0.57	0.08	S
23	Pancreatitis	7	0.50	0.07	NS
24	Reoperation for Incisional Hernia	7	0.50	0.07	S
25	Complication of Intubation	6	0.43	0.06	S
26	Epididymitis	6	0.43	0.06	NS
27	Microalbuminuria	6	0.43	0.06	NS
28	Post-operative Reintubation	6	0.43	0.06	S
29	Post-operative Renal Failure	6	0.43	0.06	S
30	Bladder Injury	5	0.36	0.05	S
31	Bowel Injury	3	0.21	0.03	S
32	Post-operative Dialysis	3	0.21	0.03	S
33	Post-operative DVT	3	0.21	0.03	S
34	Splenic Injury	3	0.21	0.03	S
35	Rhabdomyolysis	2	0.14	0.02	NS
36	Vascular Injury	2	0.14	0.02	S

This table shows 36 post-donation conditions that are found in the living kidney donors in RELIVE ImmPort used in constructing the trajectory network. They are ranked in decreasing order of number of LKDs having that condition

© 2019 Chen J et al. *JAMA Network Open.*

in the dataset. Please note that the numbers do not add up to 1,401 because each LKD can have multiple conditions after transplant. The fourth column shows the percentage of LKDs having that condition out of the 1,401 LKDs that have at least one post-donation outcome. The fifth column shows the percentage of LKDs having that condition out of the entire set of LKDs in RELIVE, including the majority of those that do not have a recorded condition. The last column denotes whether the condition is deemed a surgical complication (S) or a condition that did not arise from surgical procedure (NS).

© 2019 Chen J et al. *JAMA Network Open.*

eFigure 1. ImmPort transplant data curation workflow

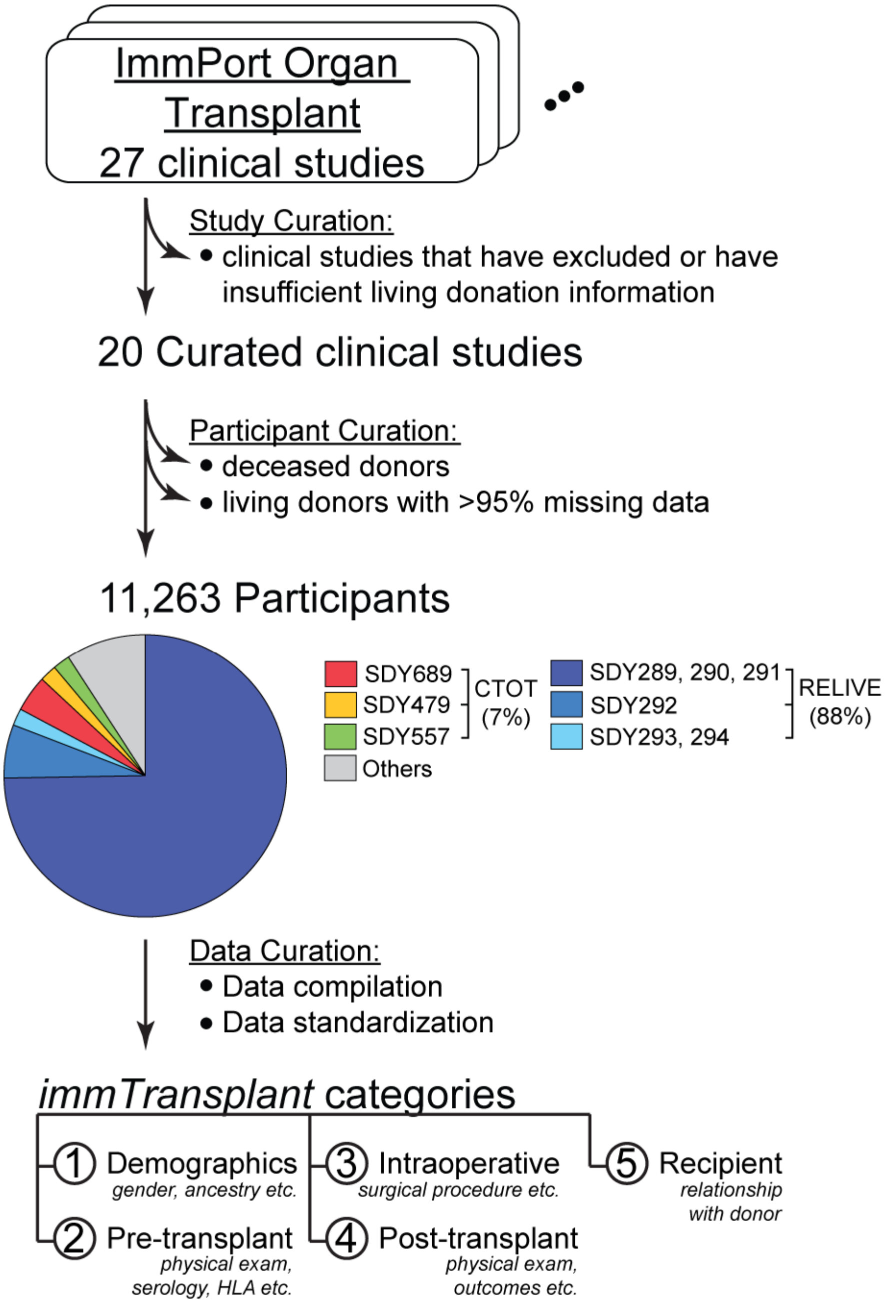

© 2019 Chen J et al. *JAMA Network Open.*

eFigure 2. Increase in female donations at age >25 years mainly due to donations to offspring and spouses

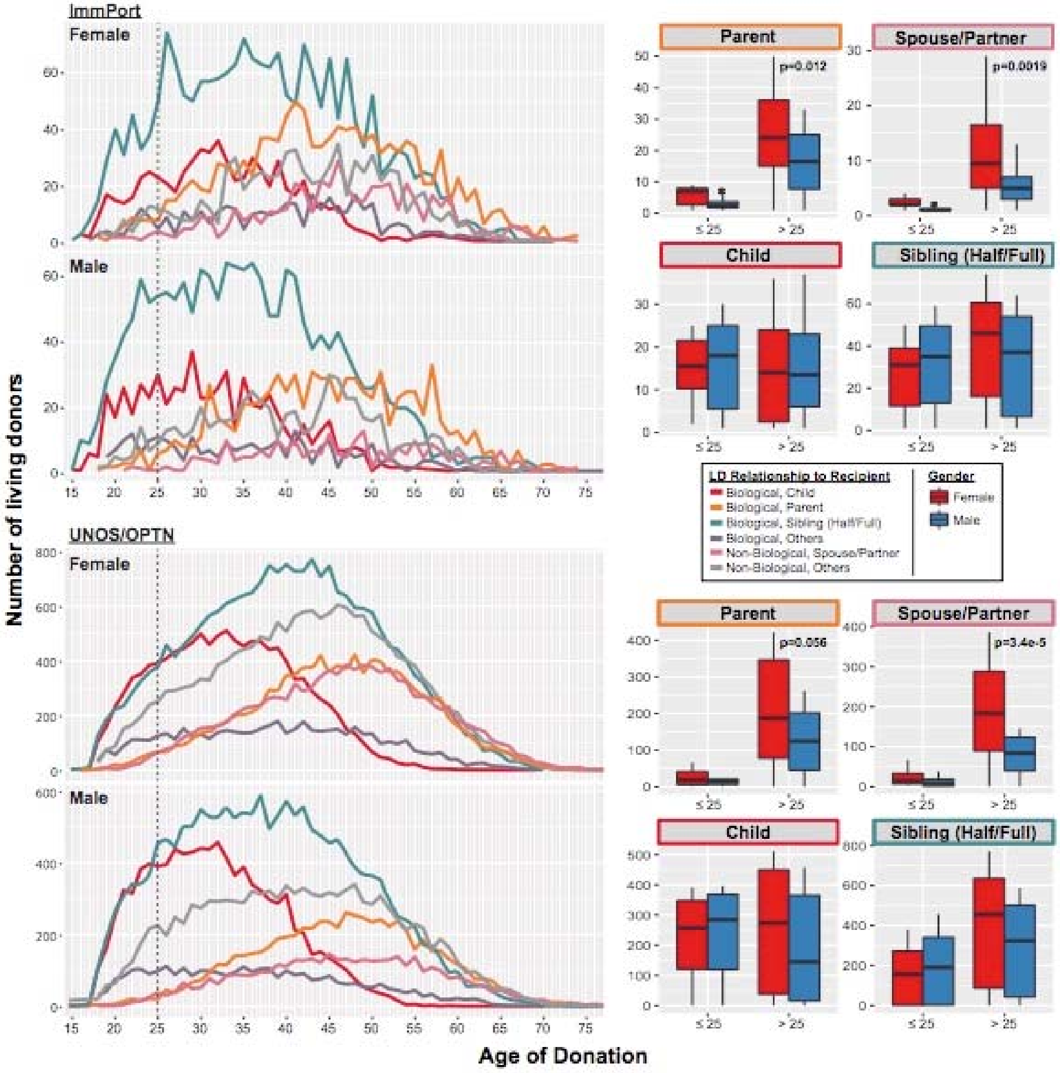

This figure compares the count distributions of the relationships of the LDs to the donors, in the ImmPort and UNOS/OPTN databases, split by gender. On the left panel, the data is being stratified by LD relationships and gender, in ImmPort and UNOS/OPTN data, between the ages 15-78. We specifically look at LKDs who are the children (red), parents (orange), siblings (green, half or full siblings included), and spouses (pink) of the transplant recipients. Other biologically-related LKDs, such as grandparents and cousins (dark grey), and other unrelated LKDs, such as friends (light grey) are separately aggregated as single categories. The right panel shows the boxplots comparing the count distributions of male (blue boxplots) and female (red boxplots) LKDs at ages ≤ 25 and > 25, for the different relationship categories. In ImmPort data, we observe statistically significant increase in female LKDs as compared to male LKDs at ages > 25, for LKDs who are parents (MW Bonferroni-corrected p=1.2e-2) and spouses (MW Bonferroni-corrected p=1.9e-3), but not in the siblings or offspring donations. In UNOS/OPTN, we also observe similar trends, in spousal living donations (MW Bonferroni-corrected statistically significant p=3.4e-5), and albeit weaker statistical evidence in LKDs who are parents (MW Bonferroni-corrected p=5.6e-2). The rest of the comparisons that do not specify p values are statistically insignificant.

© 2019 Chen J et al. *JAMA Network Open.*

eFigure 3. Higher proportions of female LKDs at age of donation >25 years

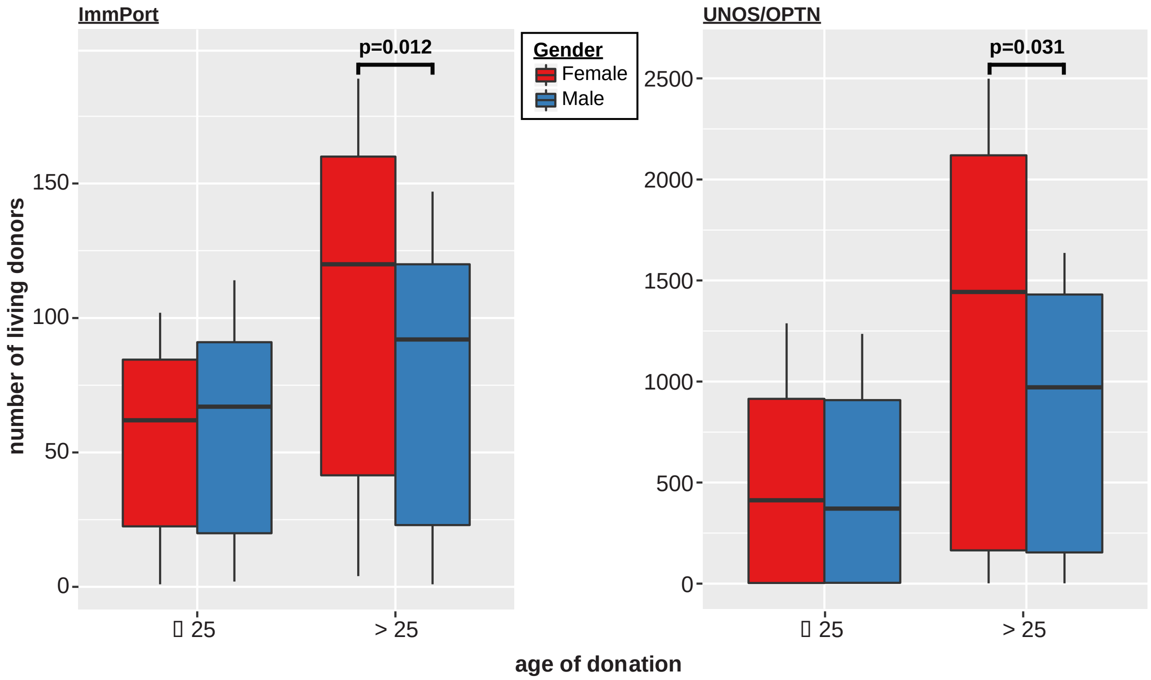

This figure shows the comparison of the count distributions of male (blue boxplots) and female (red boxplots) living donors at ages (at time of donation) ≤ 25 and > 25, for the ImmPort data (left panel) and UNOS/OPTN (right panel). In both datasets, for ages ≤ 25, there are comparable numbers of female and male living donors (Mann Whitney, or MW, tests have insignificant p values), but for ages > 25, there are more female than male living donors (MW test for ImmPort data with statistically significant Bonferroni-corrected $p = 0.012$, and for UNOS/OPTN data Bonferroni-corrected $p = 0.031$).

© 2019 Chen J et al. *JAMA Network Open.*

eFigure 4. Sex and age group bias in immTransplant and UNOS/OPTN data sets

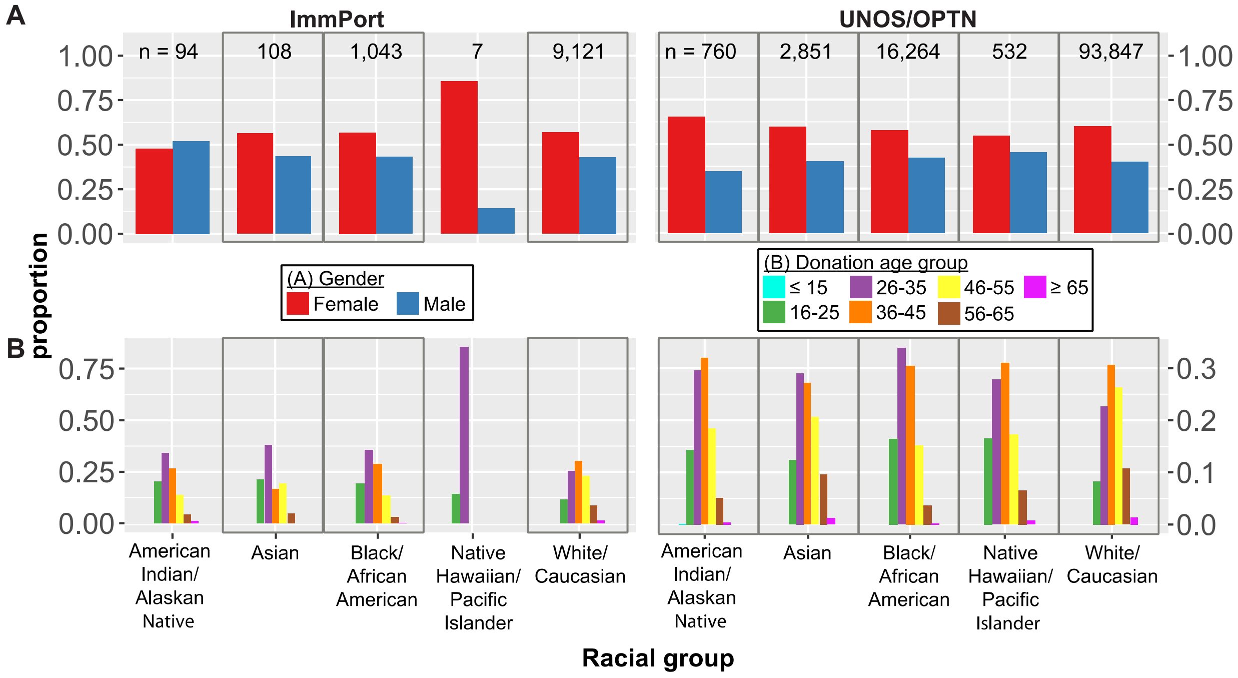

This figure shows our analyses of gender (upper panel, A) and age (lower panel, B) biases in five racial groups included in both ImmPort (left panel) and UNOS/OPTN (right panel), namely American Indian/Alaskan Native, Asian, African American, Native Hawaiian/Pacific Islander, and Caucasian/European. For the ImmPort racial groups, we only use Asian, African, and European Americans for comparisons, as the number of donors (n) in these racial groups ≥ 100 (grey boxes). We observe a female bias across all appropriate racial groups in both ImmPort and UNOS/OPTN. There is also an age group bias for donation age groups of 26-35 (purple bars in lower panel B), and 36-45 (orange bars). Note that the proportions are calculated based on the total number of LKDs within each racial group.

© 2019 Chen J et al. *JAMA Network Open.*

eFigure 5. Number and type of posttransplant conditions that can occur in each LKD

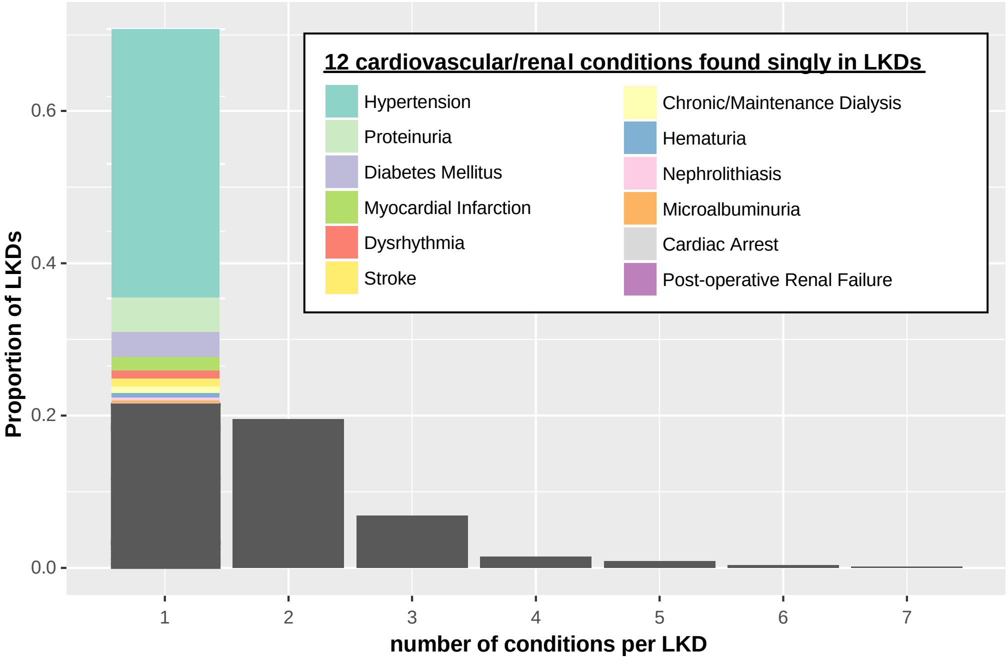

This figure shows a histogram of the number of conditions occurring for each LKD. We observe that 71% of the conditions, if they occur at least once, occur singly in each LKD after transplant, with the 12 cardiovascular- and kidney-related conditions constituting the majority of those single occurrences (70%).

© 2019 Chen J et al. *JAMA Network Open.*

eFigure 6. GFR values decrease after donation in living kidney donors

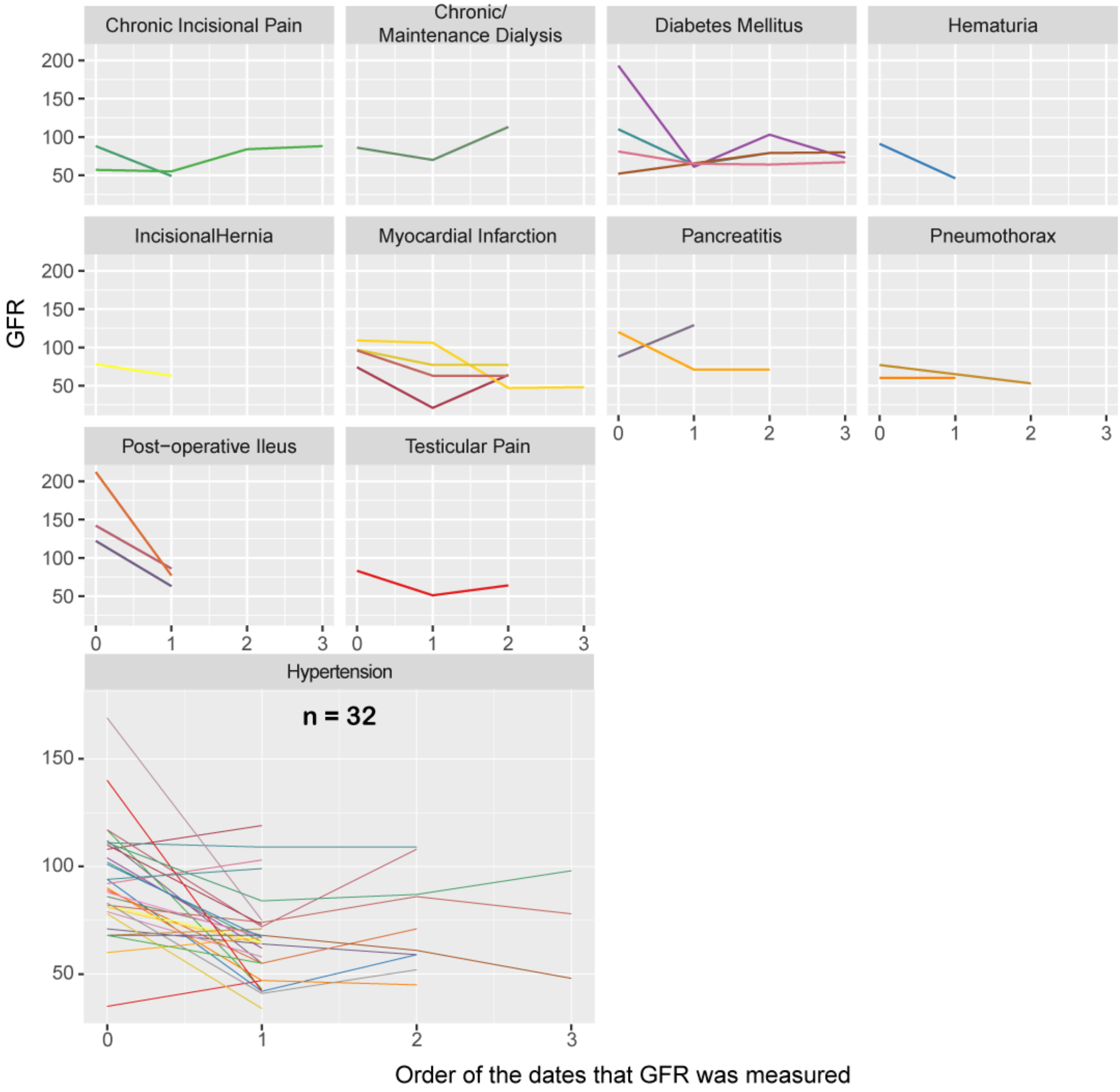

This figure shows GFR trends of LKDOs with conditions that occur singly, from pre-transplant (denoted as '0' on x-axis) and up to 3 post-transplant GFR measurements, ordered '1', '2', and '3', according to how close is the date to the date of transplantation, with '1' being the closest. Each colored line is a single LKDO with that particular condition. We observe that for most of the LKDOs, GFR values decrease with each post-transplant visit. We singled out the analysis of LKDOs with hypertension, as it has the highest number of LKDOs used in this analysis. The majority of the LKDs in this set shows a drop in GFR (25 out of 32; 78%).

© 2019 Chen J et al. *JAMA Network Open.*
